# Oleanolic Acid Attenuates Renal Fibrosis through TGF-*β*/Smad Pathway in a Rat Model of Unilateral Ureteral Obstruction

**DOI:** 10.1155/2020/2085303

**Published:** 2020-03-30

**Authors:** Dapeng Zhao, Zhongqiu Luan

**Affiliations:** The Department of Nephropathy, First Affiliated Hospital, Heilongjiang University of Chinese Medicine, No. 26 Heping Road, Xiangfang District, Harbin, Heilongjiang 150040, China

## Abstract

Renal fibrosis is a common final pathological process in the progression of kidney disease. Oleanolic acid is a bioactive pentacyclic triterpenoid and is widely found in medicinal herbs around the world. In this study, we explored the effect of oleanolic acid on renal fibrosis and the underlying molecular mechanisms by using a rat model of unilateral ureteral obstruction (UUO). Male Sprague-Dawley rats were orally administered with oleanolic acid (6 mg/kg/d) or vehicle (olive oil) for 21 days after the UUO surgery. Upon termination, urine and blood were collected for renal function analysis, and kidneys were harvested for pathological analysis by using hematoxylin-eosin and Masson trichrome staining. Changes of extracellular matrix mRNA expressions and TGF-*β*/Smad signaling in the kidneys were also determined. As a result, oleanolic acid significantly reduced the kidney index, the level of serum creatinine and blood urea nitrogen, and the urinary level of microalbumin, *α*1-microglobulin, and *N*-acetyl-*β*-glucosaminidase. Masson trichrome staining showed significantly less collagen deposition in the UUO rats with oleanolic acid treatment. Diminished mRNA expressions of collagen I, collagen III, fibronectin, and *α*-SMA in the kidney tissues were observed after the treatment. Oleanolic acid led to decreased protein expressions of TGF-*β*, TGF-*β* receptor I, and TGF-*β* receptor II, as well as the phosphorylation of Smad2. Our current study suggested that oleanolic acid could be a complementary and alternative therapy for renal fibrosis potentially by targeting the TGF-*β*/Smad pathway.

## 1. Introduction

The insidious replacement of healthy kidney structure by excessive extracellular matrix (ECM) accumulation is one of the most representative markers of kidney interstitial fibrosis [1, 2]. Epithelial-mesenchymal transition (EMT) has been demonstrated as a critical step during the development of tubulointerstitial fibrosis [3]. EMT is defined as a process that fully differentiated tubular epithelial cells undergo the transition to a secretory phenotype mesenchymal cells to promote ECM production [4, 5]. Accumulating evidence has been established that the inhibition of myofibroblasts and ECM accumulation are considered as critical means to curb the progression of kidney interstitial fibrosis.

It has been reported that TGF-*β*/Smad signaling is closely related to kidney fibrosis [6, 7]. In the canonical fibrosis related TGF-*β*/Smad signaling pathway, Smads are grouped into three classes, including (1) receptor Smads (R-Smads), Smad2, and Smad3, (2) Co-Smad, Smad4, and (3) inhibitory Smads (I-Smad), Smad7. TGF-*β* binds to its cell surface receptors and activates the serine/threonine kinase activity of R-Smads, subsequently forming a complex with Co-Smad. The complex translocates from cytoplasm to nucleus, where it regulates the transcription of target genes for fibrosis development. The I-Smad negatively regulates TGF-*β*/Smad signaling activation and functions by combining the TGF-*β* receptors and Smads for degradation via the ubiquitin-proteasome degradation mechanisms [8]. Therefore, screening and validating chemical reagents or plant natural products targeting profibrotic TGF-*β*/Smad signaling might be an effective strategy to alleviate the progression of kidney fibrosis [9].

Oleanolic acid is a natural triterpenoid in plant kingdom, medicinal herbs, and is an integral part of the human diet. Several pharmacological attributes of oleanolic acid, such as antiviral, antiinflammatory, antiangiogenic, antiapoptotic, and antitumor activities, have been reported in both *in vitro* and *in vivo* researches [10, 11]. By employing unilateral ureteral obstruction (UUO) and nephrectomy models, oleanolic acid was shown to attenuate renal fibrosis in one previous study [12], but the underlying mechanism was remained unknown. In the current work, we explored the efficacy of oleanolic acid in a UUO rat model and identified the critical role of the TGF-*β*/Smad pathway mediating the protective efficacy of oleanolic acid.

## 2. Materials and Methods

### 2.1. Materials and Reagents

Oleanolic acid was purchased from Shanghai Winherb Medical Technology Co., Ltd. It was dissolved in dimethylsulfoxide (DMSO) and diluted in olive oil for oral administration. Trizol reagent for RNA extract was purchased from Invitrogen (Carlsbad, CA). RevertAid First Strand cDNA Synthesis Kit was purchased from Thermo Scientific (Rockford, IL). Quantifast SYBR green PCR kit was purchased from QIAGEN GmbH (Valencia, CA). The primers were produced by Shanghai Sangon Biological and Technological Company. The PCR amplification was performed using the primers shown in Table 1. Antibodies including anti-TGF-*β*, anti-TGF-*β* receptor I (T*β*RI), anti-TGF-*β* receptor II (T*β*RII), anti-phosphorylated Smad2, anti-*β*-actin, and horseradish peroxidase (HRP)-labeled goat anti-rabbit or anti-mouse IgG were purchased from Cell Signaling Technology (Danvers, MA).

### 2.2. Rat Model of UUO and Animal Experimental Protocol

A total of 27 male Sprague-Dawley rats (SPF grade with weight of 220 ± 20 g) were purchased from Shanghai SLAC Laboratory Animal Co., Ltd, and grouped randomly into three groups: (1) sham group (*n* = 9), (2) UUO group (*n* = 9), and (3) oleanolic acid group (*n* = 9). All study protocols were designed by following the National Institutes of Health Guide for the Care and Use of Laboratory Animals (https://www.ncbi.nlm.nih.gov) and approved by the Institutional Animal Care and Use Committee of Heilongjiang University of Chinese Medicine. The UUO model was established for the UUO group and the oleanolic acid group. Briefly, after anaesthetized with sodium pentobarbital, rats were shaved, and an incision was made at the renal region of the left abdomen. The left ureter was exposed and ligated with 4-0 silk at two 1/3 points of the ureter without resection. Rats in the sham control group underwent a similar procedure, but without ureteral ligation. Oleanolic acid (6 mg/kg/d) and vehicle were given to rats in the oleanolic acid group and the UUO group, respectively, by oral administration. At day 21 post-UUO, all animals were sacrificed after urine collection. Blood was collected from the abdominal aorta, and the left kidneys were carefully removed and weighted. The left kidney was cut into two parts, from which one part was snap frozen in liquid nitrogen and kept at −80°C for protein and RNA extraction, and the other part was immersed into 10% neutral-buffered formalin for histopathological staining and examination.

### 2.3. Renal Function Test

The serum of rats was separated by centrifugation from blood in which serum creatinine (Scr), and blood urea nitrogen (BUN) were measured by automatic biochemical analyzer (Synchron CX5 Delta, Beckman Coulter, Miami, FL).

### 2.4. Protein Measurement in Urine

Briefly, 24-hour urine was collected before anesthesia. The level of microalbumin (MAlb) was detected by immune transmission (Hitachi7600, Hitachi, Tokyo, Japan). The level of alpha-1 microglobulin (*α*1-MG) was detected by immune rate nephelometry (protein chemistry analyzer IMMAGE800, Backman Coulter). The level of beta-2 microglobulin (*β*2-MG) was detected by microparticle immunoluminescence (AXSYM immunoassay analyzer, Abbott, Abbott Park, IL). The level of *N*-acetyl-beta-D-glucosidase (NAG) was detected by immunoturbidimetry (Synergy H4, BioTek, Winooski, VT).

### 2.5. Histologic Staining and Examination

Fixed kidney tissues were embedded in paraffin and then cut into 3 μm thick slices. The paraffin sections were stained with hematoxylin-eosin or Masson trichrome staining following the process as previously described [13]. The fibrosis score (area %) was quantitatively measured using Image Pro-Plus Software (Media Cybernetics, Silver Spring, MD) to calculate the percentage of the fibrotic area based on the color differences (green/blue).

### 2.6. RNA Preparation and Real-Time PCR

Total RNA was extracted from the frozen kidney cortex according to TRIzol protocol. Briefly, mRNA was reversely transcribed to cDNA using the RevertAid First Strand cDNA Synthesis Kit. SYBR green PCR reagent was applied for DNA amplification. The fluorescence was quantified with the PCR CFX-96 system (Bio-Rad, Hercules, CA). Relative quantification was calculated as the ratio between the amount of target template and GAPDH, which was a reference template in the same sample. The relative gene expression was calculated using the formula 2^–(ΔCt sample–ΔCt control)^ for each sample.

### 2.7. Immunoblot Analysis

Kidney cortex tissue was lysed in a lysate buffer by sonication on ice. The lysate was cleared by centrifugation at 13,000 g at 4°C for 20 min, and the supernatant was collected. Protein quantification was performed using the bicinchoninic acid protein assay kit. A total of 30 mg protein was loaded and separated by 10% SDS-PAGE gel electrophoresis and followed by transferring to the PVDF membrane in a tank. Nonspecific binding was blocked with 5% bovine serum albumin in TBS with Tween-20 for 1 h at room temperature, and then the membrane was incubated with specific primary antibodies of TGF-*β*, T*β*RI, T*β*RII, p-Smad2, Smad2, and *β*-actin at 4°C overnight. After three times washing in TBS with Tween-20, the membrane was incubated with HRP-conjugated goat anti-rabbit or anti-mouse IgG for 1 h at room temperature. Followed by TBS rinsing, protein bands were visualized using enhanced ECL and X-ray film. The ratio of target protein to GAPDH as an internal control was calculated and analyzed by Multi-analyst software program (Bio-Rad).

### 2.8. Statistical Analysis

Data were presented as mean ± standard deviations (SD). Difference between groups was analyzed by performing one-way ANOVA with Dunnett's multiple comparison test. Statistical significance was set as *P* < 0.05. Data were analyzed and graphed by Prism 6.0 (GraphPad Software, La Jolla, CA).

## 3. Results

### 3.1. Effect of Oleanolic Acid on Kidney Index and Kidney Function in the Rat UUO Model

**Figure 1:**
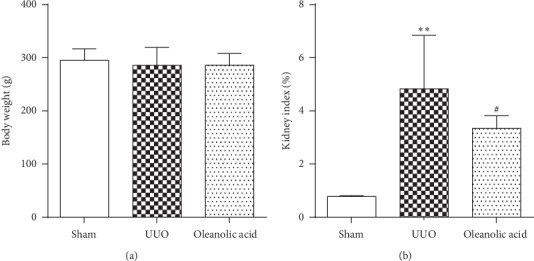
Body weight and kidney index. Oleanolic acid (6 mg/kg/d) was given to rats by oral administration for 21 days after the UUO surgery. (a) Body weight. (b) Kidney index (sham or obstructed kidney weight/body weight). Data were presented as mean ± SD. *N* = 9. ^*∗∗*^*P* < 0.01 versus sham group; ^#^*P* < 0.05 versus UUO group.

**Figure 2:**
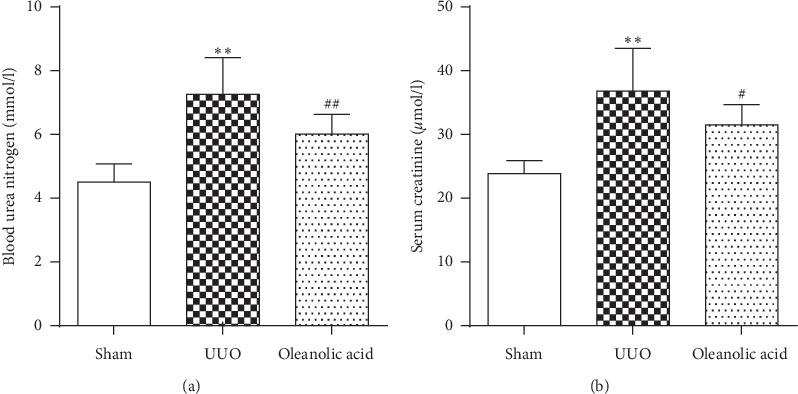
Kidney function. (a) Blood urea nitrogen (BUN). (b) Serum creatinine (Scr). Data were presented as mean ± SD. *N* = 9. ^*∗∗*^*P* < 0.01 versus sham group; ^#^*P* < 0.05 versus UUO group; ^##^*P* < 0.01 versus UUO group.

The kidney index and the levels of Scr and BUN in the UUO group were significantly increased compared with those in the sham control group (*P* < 0.01), which indicated successful modeling of UUO (Figures 1 and 2). Treatment with oleanolic acid in the UUO rats (oleanolic acid group) led to the lower kidney index, and Scr and BUN levels compared with the UUO rats treated with vehicle (UUO group, *P* < 0.05) (Figures 1 and 2). Body weight was comparable among all groups (Figure 1(a)).

### 3.2. Effect of Oleanolic Acid on Urinary Low-Molecular-Weight Protein Markers in a Rat UUO Model

**Figure 3:**
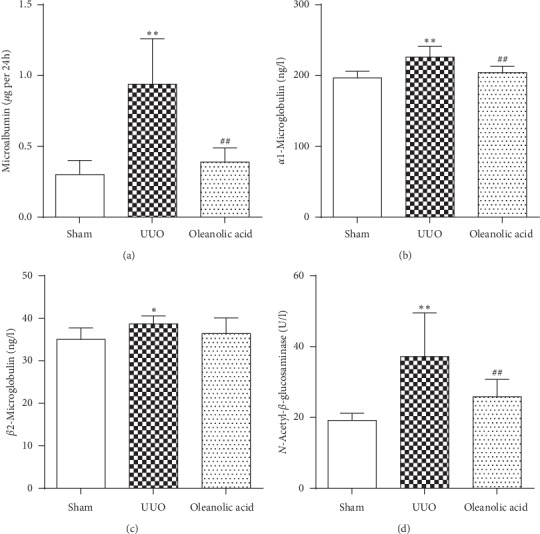
Urinary proteins. (a) 24-hour total microalbumin (MAlb). (b) Alpha1 microglobulin (*α*1-MG). (c) Beta2 microglobulin (*β*2-MG). (d) *N*-acetyl-*β*-glucosminase (NAG). Data were presented as mean ± SD. *N* = 9. ^*∗*^*P* < 0.05 versus sham group; ^*∗∗*^*P* < 0.01 versus sham group; ^##^*P* < 0.01 versus UUO group.

Urinary levels of MAlb, *α*1-MG, *β*2-MG, and NAG were significantly higher in the UUO group than in the sham control group (*P* < 0.05) (Figure 3). Treatment with oleanolic acid in the UUO rats (oleanolic acid group) led to lower urinary MAlb, *α*1-MG, and NAG levels compared with vehicle-treated UUO rats (UUO group, *P* < 0.01) (Figure 3).

### 3.3. Effect of Oleanolic Acid on Kidney Morphology in Rat UUO Model

**Figure 4:**
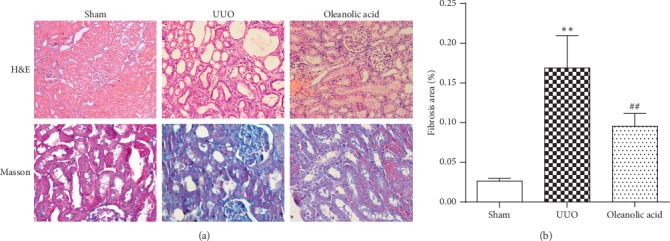
Effect of oleanolic acid on the pathological changes of kidney tubulointerstitial fibrosis induced by UUO. (a) Hematoxylin and eosin staining of kidney cortex showing the protection of oleanolic acid on renal structural damage (upper line). Masson trichrome staining of kidney cortex showing alleviating tubulointerstitial fibrosis after oleanolic acid treatment (lower line). Magnification: 400×. (b) Percentage of the Masson trichrome-positive tubulointerstitial area relative to the entire area. Data were presented as mean ± SD. *N* = 9. ^*∗∗*^*P* < 0.01 versus sham group; ^##^*P* < 0.01 versus UUO group.

Rats in the sham control group presented normal kidney morphology and regular shape of glomerulus, in addition to closely arranged renal tubules and normal epithelial cell number (Figure 4(a)). In the UUO group, the number of glomeruli in renal cortex was significantly reduced, and the periglomerular fibrosis was observed. Structure of remaining glomerular was basically normal, characterized by a large number of atrophy and expanded renal tubules, moderately widened kidney interstitium, a small amount of infiltrated inflammatory cells, and increased renal interstitium fibroblasts. The number of glomerulus in the oleanolic acid group was more than that of the model group, but the glomerulus shape was not substantially changed. However, slightly atrophied and dilated renal tubules, widened kidney interstitium, and decreased lesion were observed in the oleanolic acid group, compared with those in the model group. Additionally, UUO induced an increase of collagen on the Masson's trichrome-stained kidney sections compared with those in the sham group (*P* < 0.01, Figure 4(b)), while oleanolic acid administration resulted in significantly less collagen deposition in UUO rats (*P* < 0.01, Figure 4(b)).

### 3.4. Effect of Oleanolic Acid on ECM mRNA Expression

**Figure 5:**
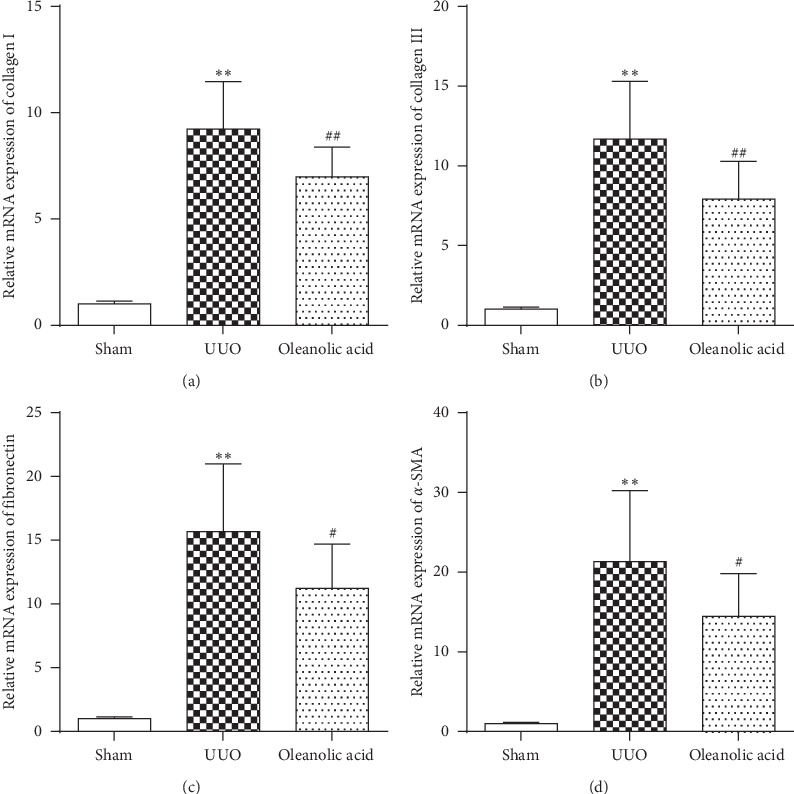
Effect of oleanolic acid on the mRNA expression of extracellular matrix. Relative mRNA expression of collagen I (a), collagen III (b), fibronectin (c), and *α*-SMA (d) were quantified by ratios to the sham group and normalized with GAPDH. Data were presented as mean ± SD. *N* = 9. ^*∗∗*^*P* < 0.01 versus sham group; ^#^*P* < 0.05 versus UUO group; ^##^*P* < 0.01 versus UUO group.

A major character of UUO-induced tubulointerstitial fibrosis is the accumulation of ECM components. Our results showed that UUO significantly increased mRNA expression of collagen I, collagen III, fibronectin, and *α-SMA* in the kidneys (*P* < 0.01, Figure 5). Intriguingly, diminished mRNA expressions of ECM components in the kidneys were observed in the oleanolic acid treated rats (*P* < 0.05, Figure 5).

### 3.5. Effect of Oleanolic Acid on Pro-Fibrotic TGF-*β*/Smad Pathway

**Figure 6:**
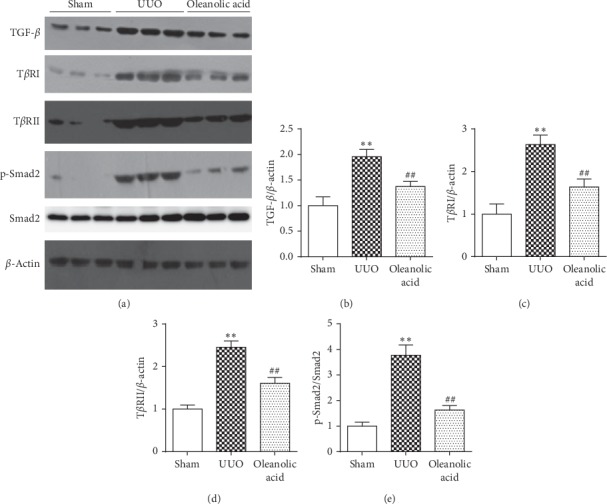
Effect of oleanolic acid on the TGF-*β*/Smad pathway. (a) Representative immunoblot image with specific antibodies against TGF-*β*, TGF-*β* receptor I (T*β*RI), TGF-*β* receptor II (T*β*RII), phosphorylated Smad2, Smad2, and *β*-actin. The expression levels of TGF-*β* (b), T*β*RI (c), T*β*RII (d), and p-Smad2/Smad2 (e) were quantified by densitometry and normalized with *β*-actin. Data are presented as mean ± SD. *N* = 9. ^*∗∗*^*P* < 0.01 versus sham group; ^##^*P* < 0.01 versus UUO group.

Increased protein expressions of TGF-*β*, T*β*RI, and T*β*RII, as well as the phosphorylation of Smad2 were observed in the UUO group (*P* < 0.01). However, the protein expressions and phosphorylation could be decreased (*P* < 0.01) after oleanolic acid treatment (*P* < 0.01, Figure 6).

## 4. Discussion

Oleanolic acid is a bioactive pentacyclic triterpenoid and is widely found in food-borne or medicinal herbs such as grapes, persimmons, blueberries, cherries, papaya, hawthorn, jujube, and loquat [14]. Previous researches revealed protective effects of oleanolic acid against diabetic nephropathy, hypertensive renal injury, renal fibrosis, polycystic kidney disease, drug-induced renal injury, heavy metal-induced renal injury, ischemia-reperfusion renal injury, and renal allograft injury [15, 16].

Several groups investigated the efficacy of oleanolic acid on renal fibrosis with different models, including mice UUO and rat nephrectomy models [12, 17]. In the current study, we validated the protective efficacy of oleanolic acid against renal fibrosis using a rat model of UUO. Rats in the UUO model group showed impaired renal function, with significantly increased BUN and Scr levels, and elevated excretion of MAlb, *α*1-MG, *β*2-MG, and NAG into the urine. H&E and Masson staining further validated the pathogenesis of kidney tubulointerstitial fibrosis induced by UUO. All these changes were attenuated by the treatment of oleanolic acid.

Tubulointerstitial fibrosis is featured by the accumulation of fibroblasts and excessive ECM deposition. Regardless of the initiating factors or pathogenic mechanisms, once the pathogenesis of chronic kidney disease is onset, healthy kidney tissue is irreversibly destroyed, and scarring becomes inevitable [18]. In this period, tubular epithelial cells and fibroblasts were activated into myofibroblasts through the process of EMT, which is partly responsible for enhanced deposition of ECM [3]. In our study, we observed significantly elevated ECM expressions induced by UUO, including collagen I, collagen III, fibronectin, and *α*-SMA as a myofibroblast marker. Importantly, we found the treatment of oleanolic acid suppressed the overexpression of ECM, which was consistent with the previous *in vitro* study, showing that oleanolic acid attenuated TGF-*β*-mediated EMT in renal tubular epithelial cells NRK-52E with downregulated expressions of *α*-SMA and fibronectin [19].

TGF-*β* is a well-characterized mediator of EMT in cultured renal tubular epithelial cells and the mice model of UUO [20, 21]. In the rat UUO model, we observed upregulated protein expressions of TGF-*β*, TGF-*β* receptor I and II. Binding of TGF-*β* to its receptors induces the formation of heteromeric receptor complex with kinase activation and leads to recruitment and phosphorylation of Smad2/3 [8]. In this study, we observed induced phosphorylation of Smad2 in the rat UUO model. Oleanolic acid reduced the expressions of TGF-*β*, T*β*RI, and T*β*RII and the phosphorylation of Smad2, suggesting oleanolic acid attenuated renal fibrosis through the TGF-*β*/Smad pathway. The phospho-Smads form heteromers with Smad4, which are subsequently transported to the nucleus where they regulate gene expressions [1]. Therefore, the future direction of this study would be the determination of the transcription activity of these Smads, and the down-signaling genes of the TGF-*β*/Smad pathway that regulated by oleanolic acid also warrant further investigation.

## 5. Conclusions

In conclusion, we reported protective effects of oleanolic acid in a UUO rat model with improved renal function and reduced ECM deposition and identified the critical role of the TGF-*β*/Smad pathway mediating the protection, suggesting oleanolic acid could be a potential complementary and alternative therapy for renal fibrosis in clinical practice. In the future work, more involved mechanisms, especially transcriptional activation of downstream promoters, need to be further clarified.

## Figures and Tables

**Table 1:** Primers used for quantitative real-time PCR.

Genes	Primers
Collagen I	Sense, 5′-TGGTGAGACGTGGAAACCTG-3′ Antisense, 5′-CTGGACCAAAAGGTGATGCTG-3′

Collagen III	Sense, 5′-AGATGCTGGTGCTGAGAAG-3′ Antisense, 5′- TGGAAAGAAGTCTGAGGAAGG-3′

Fibronectin	Sense, 5′-ACGGCACAACAGACCACCAA-3′, Antisense, 59-CAGATTTCTCAGGGGATACTTGGA-3′

*α*-SMA	Sense, 5′-GCCTATCAGAATGGGAACTACAGA-3′, Antisense, 5′-AAGCTACCATGAGGGTACTAGGAGT-3′

GAPDH	Sense, 5′-GCCTTCCGTGTTCCTACC-3′, Antisense, 5′-AGAGTGGGAGTTGCTGTTG-3′

## Data Availability

The data sets used and/or analyzed during the present study are available from the corresponding author on reasonable request.
